# Health Behavior and Metabolic Risk Factors Associated with Normal Weight Obesity in Adolescents

**DOI:** 10.1371/journal.pone.0161451

**Published:** 2016-08-25

**Authors:** Anna S. Olafsdottir, Johanna E. Torfadottir, Sigurbjorn A. Arngrimsson

**Affiliations:** 1 Research Center for Sport and Health Sciences, School of Education, University of Iceland, Reykjavik, Iceland; 2 Educational Research Institute, University of Iceland, Reykjavik, Iceland; 3 Unit for Nutrition Research, Faculty for Food Science and Nutrition, University of Iceland, Reykjavik, Iceland; Vanderbilt University, UNITED STATES

## Abstract

**Objective:**

To explore health behaviors and metabolic risk factors in normal weight obese (NWO) adolescents compared with normal weight lean (NWL) peers.

**Design and Methods:**

A cross-sectional study of 18-year-old students (n = 182, 47% female) in the capital area of Iceland, with body mass index within normal range (BMI, 18.5–24.9 kg/m^2^). Body composition was estimated via dual energy X-ray absorptiometry, fitness was assessed with maximal oxygen uptake (VO_2_max) during treadmill test, dietary intake through 24-hour recall, questionnaires explained health behavior and fasting blood samples were taken. NWO was defined as normal BMI and body fat >17.6% in males and >31.6% in females.

**Results:**

Among normal weight adolescents, 42% (n = 76) were defined as NWO, thereof 61% (n = 46) male participants. Fewer participants with NWO were physically active, ate breakfast on a regular basis, and consumed vegetables frequently compared with NWL. No difference was detected between the two groups in energy- and nutrient intake. The mean difference in aerobic fitness was 5.1 ml/kg/min between the groups in favor of the NWL group (p<0.001). NWO was positively associated with having one or more risk factors for metabolic syndrome (Odds Ratio OR = 2.2; 95% confidence interval CI: 1.2, 3.9) when adjusted for sex. High waist circumference was more prevalent among NWO than NWL, but only among girls (13% vs 4%, p = 0.019).

**Conclusions:**

High prevalence of NWO was observed in the study group. Promoting healthy lifestyle with regard to nutrition and physical activity in early life should be emphasized regardless of BMI.

## Introduction

Several adult diseases originate from lifestyle factors achieved in early- life and adulthood [1–6]. A recent study from the US demonstrated that risk factors measured in childhood (5–20 years of age) and retained in adulthood predicted cardiovascular diseases and type 2 diabetes, 26 years later [7]. High body fat, especially visceral fat can affect metabolism and increase the risk for metabolic syndrome [8], which can develop into non-alcoholic fatty liver disease [9], cardiovascular diseases and type 2 diabetes [10].

Body mass index (BMI) fails to provide information on total body fatness, therefore a specific type of obesity has been defined; normal weigh obesity (NWO), which applies to individuals with BMI within normal range (BMI, 18.5–24.9 kg/m^2^) but with excessive body fat [11]. NWO is associated with higher risk of metabolic syndrome, both in young adults [12] and older people [13]. Furthermore, high body fat is associated with higher waist circumference among adolescents [14], which is an independent risk factor, irrespective of BMI, for premature all-cause mortality [15].

Few studies have examined health behaviors associated with NWO, especially in early life. Using data on adolescents in Iceland, the objective of the study was to examine the difference in lifestyle between NWO and those within normal range of BMI with lower body fat (NWL). We further examined risk factors for metabolic syndrome between these two groups, as we hypothesized that those who were NWO were at higher risk of developing metabolic syndrome based on previous studies. Moreover we hypothesized that sedentary lifestyle and consumption of energy dense food items would be associated with NWO.

## Materials and Methods

### Study design and participants

Participants in this cross-sectional study were 18-year-old (17.7–18.9 y) high-school students from three high-schools located in the capital area of Iceland (Caucasian population). These were selected to insure a diverse group of participants by choosing schools offering both traditional academic studies and vocational education. A total of 383 students were randomly selected from the class registers and invited to participate via e-mail. Of those 275 agreed to participate (72% response rate). Since participants had to be apparently healthy and free from ailments medical history was recorded prior to participation.

Measurements for blood pressure, height, weight and waist circumference together with a face-to-face 24 hour dietary recall were conducted in each school. Aerobic fitness was measured in the laboratory on a second day and body composition and bone mineral density was examined with dual energy X-ray absorptiometry (DXA) at the Icelandic Heart Association on a third day. A fourth day was chosen for taking fasting blood samples in the morning; these were taken at the schools. During the course of the study participants answered questionnaires on health behaviors over the internet. Each participant finished all measurements within a span of 7–10 days. The study protocol including consent procedure was approved by the Icelandic Ethical Review Board (VSN07-125-S1), the Icelandic Data Protection Authority (S3612/2008) and the Icelandic Radiation Protection Institute (29092008–26). The principals in the high schools involved, gave permission to contact the students. Written informed consent was obtained from all study participants.

### Measurements

Blood pressure was measured with electronic monitor (ADC Advantage 6013) and the mean value of three measurements with one minute rest in between was used. Anthropometric measurements were taken with participants wearing light clothing and without shoes, using a standard protocol. Height was measured to the nearest 0.1 cm (Seca 206; Hamburg, Germany) and weight to the nearest 0.1 kg using portable electronic scale (Seca 799). Body mass index was calculated as weight in kilograms divided by height in meters squared (kg/m^2^). Waist circumference was measured to the nearest 0.1 cm at the narrowest place between the iliac crest and the bottom of the rib cage with a spring loaded inelastic measuring tape. Body composition and bone mineral density were estimated using DXA (GE Lunar IDXA software 11.40.004) after removing all metal objects.

Dietary intake was assessed by face-to-face 24-hour recalls The method was originally designed for the Icelandic national dietary survey and built on a telephone interview, which has been validated; it consists of four steps as has been described earlier [16]. For quantification, participants received a booklet with four portion sizes of 49 dishes or foods, and pictures of standard household equipment in different sizes. All data were entered directly into an interview-based nutrient calculating program, ICEFOOD, based on the National Nutrition Database, ISGEM. Nutrient losses due to food preparation were included in the calculations. Questionnaires on lifestyle and health behaviors offered additional data on dietary habits as well as information on physical activity, sedentary behaviors, smoking, alcohol use and sleep.

To assess aerobic fitness, maximal oxygen uptake (VO_2_max) was assessed using open circuit spirometry (Parvomedics Trumax 2400; Sandy, UT, USA) with a graded exercise test protocol on a treadmill (Quasar med, HP Cosmos; Traunstein, Germany) as described previously [17, 18]. Briefly, participants ran at constant speed (8–13 km/h) depending upon fitness and the grade of the treadmill was increased 2% every two minutes until volitional exhaustion. Expiratory air was collected (30 s averages) and oxygen consumption and carbon dioxide production calculated and used to calculate the respiratory exchange ratio. Heart rate was measured continuously with a heart rate monitor (Polar), and at the end of each stage, the participants rated their perceived exertion (Borg scale 6–20) [19].

Blood samples were drawn early in the morning after an overnight fast (>12 hours) and analyzed at Landspitali University Hospital for serum total cholesterol (TC), high-density lipoprotein (HDL) cholesterol, triglycerides (TG), glucose and insulin. Low-density lipoprotein (LDL) cholesterol was then calculated by using the equation by Friedewald et al. [20]. The homeostasis model assessment of insulin resistance (HOMA2-IR) was used as an index to estimate insulin resistance. The updated computer HOMA2-IR model with nonlinear solution using the 75th quartile as a cutoff was used to define high HOMA2-IR (http://www.dtu.ox.ac.uk/homacalculator/index.php) [21].

### Outcome assessment—Normal Weight Obesity (NWO)

The subjects whose BMI was 18.5 to 24.9 kg/m^2^ and with percentage body fat above 17.6% in males and above 31.6% in females were defined as NWO according to Lohman’s criteria [22]. Height and weight were measured in 274 subjects and of those 196 (72%) were within normal BMI. For participants with normal BMI, information on body fat was missing for 14 subjects, leaving 182 in the analysis.

### Statistical analysis

In all statistical analyses, we used SPSS software, version 22.0 (SPSS Inc., 2009, IBM Chicago, IL, www.spss.com). Differences in mean values of nutrient intake and anthropometric- and metabolic parameters according to the presence or absence of NWO were tested by the Student t-test (adjusted for sex) when variables had a normal distribution or by the Mann-Whitney nonparametric test otherwise. Statistical differences in categorical variables were calculated using the chi-square test. When examining the components used to define metabolic syndrome according to the presence or absence of NWO we used the definition from the Joint Interim Statement (JIS) of the IDF Task Force on Epidemiology and Prevention, National Heart, Lung and Blood Institute, American Heart Association, World Heart Federation, International Atherosclerosis Society and International Association for the Study of Obesity [23]. When evaluating some of the component used for the criteria of metabolic syndrome high waist circumference was defined as ≥94 cm for men and ≥80 cm for women, low HDL-cholesterol was defined as <40 mg/dL for men and <50 mg/dL for women, high triglycerides was defined as ≥150 mg/dL, high blood pressure was defined as systolic pressure ≥130 mmHg and/or diastolic pressure ≥85 mmHg and high fasting blood glucose was defined as ≥100 mg/dL [23].

Logistic regression models were used to calculate odds ratios (ORs) and 95% confidence intervals (CIs) for having one or more risk factors for metabolic syndrome using NWO as the explanatory variable. Due to the cross-sectional design of the study and the fact that all participants were in the same age, we only used sex as an adjustment variable in our final model. Since BMI could lie in the causal pathway for developing metabolic syndrome we omitted this variable from the final regression analysis but all participants in the model had BMI within the normal weight range. Lifestyle variables collected and explored as risk factors for NWO in the present study were also not included in the model, since they are probably also intermediate steps in affecting the development of metabolic syndrome and therefore, acting as mediators rather than true confounders. However, there were few lifestyle factors that were different between the groups and for secondary analysis we explored the association when adjusting for sex, calorie intake (continuous), breakfast skipping (yes/no), sleeping enough (yes/no) and aerobic fitness (continuous).

## Results

### Overall findings

In total 274 subjects had measures for height and weight. The mean BMI (±SD) was 23.0 ± 3.8 kg/m^2^ and 5% were underweight, 72% normal weight, 16% overweight and 7% obese. Those with normal weight and information on body composition (n = 182) were subjects of the present study and among them, 42% (n = 76) were defined as NWO.

### Lifestyle

Characteristic and lifestyle factors were compared between those defined as NWO and those that were normal weight but below the criteria for body fat (NWL) that was chosen to define NWO (Table 1). About 61% of the NWO group consisted of males, and in total 35% of the normal weight females were NWO compared with 48% of the males. Fewer NWO participants were physically active (p = 0.014), ate breakfast (p = 0.015), and consumed vegetables more than 4 times per week (p = 0.008) compared with NWL. No difference was seen in the frequency of consumption of other food items such as soda drinks, sweets, fast food, fruits and fish between the two groups. However, a borderline significance was found for less frequent use of fish oil supplements among NWO compared with NWL (p = 0.057). Furthermore a high proportion of participants in both groups reported that they usually did not get enough sleep during the night; 83% in the NWO group and 72% in the NWL group (p = 0.100).

**Table 1:** Characteristic of the 18-year-old participants and lifestyle factors.

Variables	Normal weight obesity^a^		p
	Yes (n = 76)	No (n = 106)	
	n (%)	n (%)	
Sex			
- Women	30 (39.5)	56 (52.8)	0.075
- Men	46 (60.5)	50 (47.2)	
Smoking history			
- Never/former	55 (75.3)	84 (80.0)	0.460
- Current	18 (24.7)	21 (20.0)	
Alcohol consumption			
- Yes	70 (93.3)	92 (87.6)	0.208
Physical activity^b^			
- Sedentary	21 (28.0)	16 (15.2)	0.014
- Medium active	25 (33.3)	26 (24.8)	
- Very active	29 (38.7)	63 (60.0)	
Getting enough sleep			
- No	62 (82.7)	75 (72.1)	0.100
Daily dietary pattern			
- Breakfast	42 (56.0)	77 (73.3)	0.015
- Lunch	65 (86.7)	87 (82.9)	0.487
- Dinner	67 (89.3)	97 (92.4)	0.479
- Evening snack	18 (24.0)	24 (22.9)	0.858
Sugary soda drinks			
- ≥ 1 times per week	55 (75.3)	81 (77.1)	0.781
Sugar free soda drinks			
- ≥ 1 times per week	32 (43.8)	43 (41.0)	0.702
Fruits			
- ≥ 4 times per week	43 (58.9)	63 (60.0)	0.884
Vegetables			
- ≥ 4 times per week	27 (37.0)	60 (57.1)	0.008
Sweets			
- ≥ 4 times per week	25 (33.8)	47 (44.8)	0.140
Fast food			
- ≥ 2 times per week	33 (45.2)	44 (41.9)	0.662
Fish oil supplement			
- Yes	25 (34.2)	51 (48.6)	0.057
Fish meals			
- ≥ 2 times per week	35 (47.9)	60 (57.1)	0.226

^a^ Normal weight obesity defined as a BMI from 18.5 to 24.9 kg/m2 and percent body fat above 17.6% in males and above 31.6% in females, measured by DXA (dual energy X-ray absorptiometry). Those who are not normal weight obese are within the normal range of BMI and have body fat below that defined for normal weight obesity.

^b^ Self-reported physical activity according to questionnaire

All P values were calculated by the chi-square test.

Numbers may not add up to total because of missing values.

### Energy- and nutrient intake

Calculated energy- and nutrient intake using the 24-hour recall method revealed no significant difference in intake between the two groups although there was a borderline difference (p = 0.059) in iron intake among females, where the NWO group had on average lower intake than NWL (Table 2).

**Table 2:** Daily energy and nutrient intake using 24-hour dietary recall among 18-year-old students.

Variables	Normal weight obesity^a^		p
	Yes (n = 71)	No (n = 103)	
	Mean ± SD	Mean ± SD	
Energy intake			
- Kcal	2559 ± 1108	2486 ± 921	0.989
- KJ	10711 ± 4639	10402 ± 3817	0.989
- Males (kcal)	3019 ± 1123	2870 ± 930	0.805
- Females (kcal)	1931 ± 720	2163 ± 767	0.166
% of energy from protein	17.2 ± 5.1	17.3 ± 5.1	0.710
- Males	17.8 ± 5.1	18.3 ± 4.9	0.318
- Females	16.4 ± 4.9	16.5 ± 5.1	1.000
% of energy from fat	31.2 ± 9.3	32.6 ± 8.5	0.263
- Males	31.6 ± 8.8	32.1 ± 9.2	0.795
- Females	30.6 ± 10.1	33.1 ± 7.6	0.187
- *Saturated*	*12*.*4 ± 4*.*1*	*13*.*5 ± 4*.*6*	*0*.*146*
- *Monounsaturated*	*10*.*0 ± 4*.*2*	*10*.*0 ± 3*.*1*	*0*.*500*
- *Polyunsaturated*	*4*.*4 ± 2*.*6*	*4*.*4 ± 2*.*2*	*0*.*523*
- *Omega-3 fatty acids*	*0*.*15 ± 0*.*3*	*0*.*20 ± 0*.*4*	*0*.*496*
% of energy from CHO	50.7 ± 9.2	49.0 ± 8.8	0.221
- Males	49.5 ± 7.3	48.6 ± 9.7	0.347
- Females	52.4 ± 11.1	49.3 ± 8.0	0.319
- *Added sugar*	*10*.*9 ± 6*.*8*	*11*.*5 ± 6*.*9*	*0*.*701*
- *Alcohol*	*0*.*51 ± 2*.*6*	*0*.*88 ± 4*.*4*	*0*.*729*
Dietary fiber (g)	19.4 ± 8.9	19.9 ± 8.6	0.397
- Males	21.4 ± 10.1	20.5 ± 7.5	0.861
- Females	16.3 ± 8.1	18.8 ± 8.4	0.116
Vitamin A (μg-RAE)^b^	943 ± 795	1343 ± 2083	0.314
- Males	1064 ± 775	1630 ± 2350	0.247
- Females	778 ± 806	1152 ± 1903	0.483
Retinol (μg)	639 ± 507	1034 ± 1814	0.135
- Males	752 ± 570	1349 ± 2327	0.151
- Females	484 ± 360	825 ± 1381	0.259
Vitamin D (μg)	5.3 ± 6.4	7.4 ± 11.2	0.361
- Males	6.3 ± 6.9	7.5 ± 8.4	0.451
- Females	3.9 ± 5.4	6.8 ± 12.7	0.526
Vitamin E (mg)	13.6 ± 12.7	14.1 ± 16.0	0.756
- Males	16.4 ± 14.7	15.1 ± 13.8	0.675
- Females	9.8 ± 8.1	12.9 ± 17.2	0.844
Total folate (μg-DFE)	439 ± 306	450 ± 268	0.531
- Males	501 ± 307	503 ± 263	0.799
- Females	355 ± 288	396 ± 257	0.248
Vitamin C (mg)	207 ± 234	169 ± 166	0.612
- Males	221 ± 231	163 ± 169	0.238
- Females	188 ± 240	167 ± 160	0.811
Calcium (mg)	1373 ± 713	1335 ± 743	0.673
- Males	1616 ± 703	1681 ± 867	0.808
- Femals	1040 ± 587	1022 ± 484	0.918
Iron (mg)	16.1 ± 10.7	19.7 ± 19.4	0.224
- Males	18.4 ± 9.1	19.4 ± 10.1	0.741
- Females	13.1 ± 12.2	19.5 ± 24.3	0.059
Selenium (μg)	80.9 ± 43.5	79.4 ± 43.6	0.742
- Males	95.2 ± 44.0	95.3 ± 49.5	0.905
- Females	61.5 ± 35.1	65.1 ± 31.5	0.462
Iodine (μg)	142 ± 101	170 ± 142	0.328
- Males	151 ± 92	210 ± 163	0.151
- Females	129 ± 113	135 ± 120	0.462

Abbrevations: SD, standard deviation; CHO, carbohydrates.

^a^Normal weight obesity defined as a BMI from 18.5 to 24.9 kg/m2 and percent body fat above 17.6% in males and above 31.6% in females, measured by DXA (dual energy X-ray absorptiometry).

P values were calculated by the Student t-test for percent of energy for fat and saturated fat. Overall means were adjusted for sex. All other P values were calculated using the Mann-Whitney U test.

^b^As retinol activity equivalents

### Anthropometry, fitness and metabolic parameters

Those in the NWO group were on average 5.1 kg heavier (p <0.001), with 1.3 kg/m^2^ higher BMI (p <0.001), had 5.5 cm greater waist circumference (p <0.001) and 6.2 percentage points higher body fat (p <0.001) than NWL (Table 3). The mean difference between the groups in aerobic fitness was 3.4 ml/kg/min favoring the NWL group. When adjusted for sex, the mean difference in aerobic fitness increased to 5.1 ml/kg/min (p <0.001). There was no difference between the groups in blood pressure, or bone mineral density. With regard to metabolic parameters females with NWO had a marginally significant tendency for higher values of low density lipoproteins than NWL females (p = 0.069). Higher serum insulin and HOMA-2 insulin resistance was also observed among NWO compared with NWL (p = 0.004 and p = 0.003, respectively). There was no difference between the groups in high density lipoproteins, triglycerides, and blood glucose.

**Table 3:** Normal weight obesity according to anthropometric, fitness and metabolic parameters– 18-year-old students.

Variables	Normal weight obesity^a^		p
	Yes (n = 76)	No (n = 106)	
	Mean ± SD	Mean ± SD	
Height (cm)	179.9 ± 9.2	175.5 ± 9.3	0.254
- Males	181.9 ± 7.7	182.7 ± 7.1	0.679
- Females	169.2 ± 4.8	169.0 ± 5.3	0.744
Weight (kg)	70.0 ± 9.1	64.9 ± 8.4	<0.001
- Males	73.7 ± 9.1	70.5 ± 7.7	0.046
- Females	64.3 ± 5.7	59.9 ± 5.2	0.001
BMI (kg/m^2^)	22.3 ± 1.8	21.0 ± 1.5	<0.001
- Males	22.2 ± 1.8	21.2 ± 1.5	0.001
- Females	22.5 ± 1.8	21.0 ± 1.5	<0.001
Waist circumference (cm)	79.2 ± 5.5	73.7 ± 4.6	<0.001
- Males	80.3 ± 5.4	76.0 ± 3.5	<0.001
- Females	77.5 ± 5.1	71.7 ± 4.7	<0.001
Body fat (%)	26.9 ± 7.2	20.7 ± 7.2	<0.001
- Males	21.8 ± 3.9	13.7 ± 2.2	<0.001
- Females	34.7 ± 2.4	27.0 ± 3.3	<0.001
Bone mineral density (g/cm^2^)	1.23 ± 0.11	1.23 ± 0.11	0.167
- Males	1.27 ± 0.11	1.28 ± 0.11	0.418
- Females	1.16 ± 0.08	1.19 ± 0.10	0.245
Aerobic fitness, VO_2_ (ml/kg/min)	45.9 ± 8.2	49.3 ± 8.2	<0.001
- Males	50.9 ± 5.7	55.8 ± 5.5	<0.001
- Females	37.8 ± 4.3	43.2 ± 4.9	<0.001
High density lipoprotein (mg/dL)	55.3 ± 11.6	58.0 ± 13.5	0.236
- Males	51.1 ± 11.0	50.3 ± 9.2	0.772
- Females	61.3 ± 10.1	64.8 ± 13.0	0.411
Low density lipoprotein (mg/dL)	93.1 ± 22.7	88.7 ± 20.9	0.143
- Males	89.6 ± 21.2	88.1 ± 18.3	0.620
- Females	98.6 ± 24.1	89.3 ± 23.0	0.069
Triglycerides (mg/dL)	94.4 ± 43.2	83.4 ± 32.9	0.119
- Males	92.1 ± 45.1	79.6 ± 31.2	0.188
- Females	98.1 ± 40.7	86.7 ± 34.2	0.238
Systolic blood pressure (mmHg)	120.6 ± 11.0	120.3 ± 9.5	0.370
- Males	123.8 ± 10.8	124.0 ± 9.8	0.928
- Females	115.8 ± 9.6	118.2 ± 8.9	0.235
Diastolic blood pressure (mmHg)	70.1 ± 7.3	70.5 ± 6.4	0.565
- Males	70.8 ± 7.3	70.2 ± 7.1	0.706
- Females	69.0 ± 7.3	70.7 ± 5.8	0.149
Blood glucose (mg/dL)	83.6 ± 14.4	80.9 ± 5.8	0.119
- Males	85.2 ± 17.9	82.2 ± 5.8	0.670
- Females	81.1 ± 5.8	79.7 ± 5.7	0.217
Serum Insulin (μU/mL)	9.0 ± 5.8	6.7 ± 2.8	0.004
- Males	9.2 ± 6.8	6.5 ± 2.5	0.054
- Females	8.8 ± 3.7	6.9 ± 3.1	0.023
HOMA-2 insulin resistance	1.6 ± 1.0	1.2 ± 0.5	0.003
- Males	1.7 ± 1.2	1.2 ± 0.4	0.052
- Females	1.6 ± 0.6	1.2 ± 0.6	0.020

^a^Normal weight obesity defined as a BMI from 18.5 to 24.9 kg/m2 and percent body fat above 17.6% in males and above 31.6% in females, measured by DXA (dual energy X-ray absorptiometry).

P values were calculated by the Student t-test for systolic blood pressure, aerobic fitness and bone mineral density. Overall means were adjusted for sex. All other P values were calculated using the Mann-Whitney U test.

### Metabolic Disorders

Five participants (2.7%) had metabolic syndrome according to the JIS, four of whom had NWO (Table 4). The prevalence of having one risk factor for developing metabolic syndrome was higher among NWO compared with NWL (42% vs. 26%, p = 0.019). In total 58% of the subjects with NWO had one or more risk factors for metabolic syndrome while 39% of NWL were in the same position (p = 0.010). Adjusting for sex, NWO was positively associated with a 2.2 fold increased risk of having one or more risk factors for metabolic syndrome (OR = 2.2; 95% CI: 1.2, 3.9) compared with NWL. When further adjusted for some of the key factors that stood out in the lifestyle (breakfast skipping, sleeping enough and aerobic fitness) among the study participants as well as sex and calorie intake, we found the risk of having one or more risk factors for metabolic syndrome still to be twofold among the NWO group (95% CI: 1.0, 4.1) with a borderline significance (p = 0.067) compared to the NWL group. When examining the prevalence within the cut points for each component used in the definition of metabolic syndrome, we only observed a statistically significant difference for high waist circumference; the prevalence being higher among those with NWO (p = 0.019). High waist circumference was only observed among females in the study, with 10 out of 14 being in the NWO group.

**Table 4:** Prevalence of metabolic syndrome and its components among 18-year-old students who are normal weight obese or normal weight lean.

Variables	Normal weight obese^a^ (n = 76)	Normal weight lean (n = 106)	p^b^
	n (%)	n (%)	
Metabolic syndrome—JIS^c^			0.019
- Zero risk factor	32 (42)	65 (61)	
- One risk factor	32 (42)	27 (26)	
- Two risk factors	8 (11)	13 (12)	
- Three risk factors	4 (5)	1 (1)	
High waist circumference^d^	10 (13)	4 (4)	0.019
Low HDL-cholesterol^e^	5 (7)	8 (8)	0.802
High Triglycerides^f^	6 (8)	4 (8)	0.235
High blood pressure^g^	17 (22)	21 (20)	0.676
High blood Glucose^h^	2 (3)	1 (1)	0.382
HOMA2-IR^i^ ≥1.8	20 (26)	18 (17)	0.135

^a^Normal weight obesity defined as a BMI from 18.5 to 24.9 kg/m2 and percent body fat above 17.6% in males and above 31.6% in females, measured by DXA (dual energy X-ray absorptiometry).

^b^P value calculated by the chi-square test.

^c^Metabolic syndrome defined according to the Joint Interim Statement (JIS) of the IDF Task Force on Epidemiology and Prevention, National Heart, Lung and Blood Institute, American Heart Association, World Heart Federation, International Atherosclerosis Society and International Association for the Study of Obesity.

^d^High waist circumference (≥94 cm for men and ≥80 cm for women).

^e^Low HDL-cholesterol (<40 mg/dL for men and <50 mg/dL for women).

^f^High triglycerides (≥150 mg/dL).

^g^High blood pressure (systolic pressure ≥130 mmHg and/or diastolic pressure ≥85 mmHg).

^h^High fasting blood glucose (≥100 mg/dL).

^i^Cut-off point for high HOMA2-IR was set above the 75th quartile.

## Discussion

In the present study, high prevalence (42%) of normal weight obesity among 18-year-old Icelandic high school students was observed. These findings are based on having BMI within normal range and Lohman’s criteria [22] of percentage body fat above 17.6% in males and 31.6% in females, using DXA to assess body composition. NWO was also found to be associated with less desirable health behaviors and increased metabolic risk.

Surprisingly, NWO was more common among males in our study, opposite to findings from the earliest research on NWO that exclusively considered girls to be affected [11] and a study from Switzerland revealed NWO to be almost non-existent in men [24]. Diverse terms and definitions have been used for NWO and different cut-off points for body fat percentage have been suggested. However, there is currently no consensus on how to define excess fatness by percent body fat [25] and the effect of chosen cut-offs and criteria used has an influence on perceived prevalence of NWO showing prevalence between 5–45% [12, 26, 27]. The cutoffs based on Lohman’s criteria [22] have however been widely accepted and used by others in body composition research where the need for empirical cutoffs has risen [18, 28]. The type of measurement used for assessing body fat may also impact the results since skinfold thickness measurements have been shown to systematically underestimate body fat percentage when compared with DXA[29]. A recent study from Brazil reported only 9% prevalence of normal weight obesity among 23–25 years old participants, but they used skinfolds to estimate body fat and chose higher cut points for body fat to classify NWO [12]. Another study where DXA was used and body fat percentage was divided into sex-specific tertiles showed that already at what was defined as medium body fat (15.3–20.7% in men and 29.8–34.9% in women) metabolic abnormalities were evident [30]. These findings indicate the importance of lower cut-offs for NWO.

In our study, NWO was associated both with lower fitness, measured as VO_2_max, and with lower physical activity levels according to self-reported data from participants. While NWO studies with actual fitness measures are few, physical inactivity has been suggested to be associated with NWO in former studies [12, 25, 31]. Fewer studies have investigated the associations between dietary intake and NWO and, in agreement with our findings, an association has seldom been found with either amount of energy, the proportion of macronutrients or single nutrients [31, 32]. There are two exceptions. First, a study among Korean adults, in which very high carbohydrate diet (≥73.9% of energy compared with <59.9%), and particularly when consumed as snack, was positively associated with metabolically obese normal weight participants (MONW) and high protein diet was found to be preventive (≥17.1% of energy from protein compared with <12.2%) [33]. Apart from the slightly different definitions of NWO and MONW, this macronutrient composition is far from what is common in the other studies with a more westernized diet. Second, the Brazilian study of Madeira et al. [12] only considered the percentage of energy from fat in the diet and found it to be higher among NWO than NWL young adults.

Considering the quality of diet in our study, both groups were on average slightly above the recommendations for consumption of saturated fat and added sugar, which is recommended to be below 10% of the total energy intake. Both groups were also on average below the Icelandic recommendation for fiber- and vitamin D intake and the NWO group was on average below the recommendations for selenium intake. Looking at food choices the NWO participants were eating vegetables less regularly and there was a trend towards less prevalent use of fish oil supplements, which may indicate a generally lower intake of both omega-3 fatty acids, vitamin D, and fiber among the NWO group despite not being observed from the 24-hour recalls. Correspondingly, in Finland low intakes of healthy foods such as root vegetables, cereals and fish were observed among NWO individuals along with higher consumption of unhealthier choices. The authors also suggested low fibre intake as a potential risk factor for NWO [31]. Considering physical activity and nutrition as opposites of energy balance the findings of the present study, with NWO consuming similar amounts of energy as NWL while engaging in less physical activity as well as being less fit and having less preferable eating habits, are in line with suggestions of the importance of higher energy throughput to maintain leanness [34]. Energy balance may be easier to achieve when energy expenditure is higher compared with a lower energy expenditure where food restriction may be required to keep the balance [35].

In our study, only 56% of the NWO participants reported eating breakfast on a daily basis, compared with 73% of their NWL peers. Yet, both groups consumed, on average, the same amount of calories suggesting that missing a breakfast might lead to excess hunger or rebound overeating [36] resulting in consumption of large portion sizes at following meals. A large study from the US has reported that breakfast skipping children and adolescents have higher BMI z-scores, waist circumference and prevalence of obesity compared with breakfast consumers [37]. These results are similar to ours with regard to waist circumference, an important risk factor for premature all-cause mortality especially at younger ages [15], as those defined with NWO had on average higher waist circumference than their NWL counterparts.

The growing number of research considering NWO and metabolic risk factors in recent years has led to general acceptance and concern over the metabolic dysregulation found among people classified as NWO [25]. Likewise, our results showed that even at young age, metabolic risk was considerably higher among NWO participants compared with the NWL group, with high waist circumference being most alarming. Even though the participants included in this study were all within the range considered as normal-weight according to BMI, the difference in average BMI between NWO and NWL was notable. By adjusting for BMI, the risk of having one or more risk factors for metabolic syndrome diminished. Body composition of persons with BMI values close to 25 kg/m^2^ (25–27 kg/m^2^ for men and 24–26 kg/m^2^ for women) may commonly be misclassified and it has been suggested that simple formula based on a single DXA scan could be used for evaluating fatness of individuals instead of relying on BMI in clinical practice [38].

### Strengths and limitations

The precise laboratory measures we used to assess fatness and fitness support the validity of the findings. Our study is however limited by the cross-sectional design, which does not allow for the determination of cause and effect relationships. Also, the present study is affected by relatively few participants, but it must be stated that the students came from three high schools located in the capital area of Iceland. There are 31 high schools in total in Iceland and according to Statistics Iceland (http://www.statice.is/), there were 4.623 people who were 18 years old living in Iceland at the time of study recruitment. With 268 students participating in the present study, we have almost 6% of the population of 18-year-olds living in the country. Furthermore, the study sample reasonably reflects 18-year-old adolescent in Iceland since it was recruited from metropolitan Reykjavik where 67% of all Icelandic high-school students are enrolled and all academic structures are presented. One of the strengths of our study is that all participants are the same age, which eliminates the confounding effect of age and many other age-dependent covariates, but the rate of NWO increases with age[13, 39]. Furthermore, this age group was chosen since adolescents in Iceland formally gain independence from their parents/caregivers at 18 years and that may be associated with more rapid changes in health behaviors than in prior years. In many other countries this age often means the end of high-school and leaving home with increased autonomy in decision-making [40]. Generally, adolescence is a period characterized by changes in body composition and health behaviors [41] and studies on NWO at this age are lacking.

## Conclusion

The concept of NWO has recently gained attention since it is an important risk factor for cardiovascular disease and metabolic dysregulation, but it has yet to be recognized and included in clinical practice [42]. If BMI is used as the only measure of adiposity, those with NWO will go undiagnosed despite having an increased cardiovascular disease risk. BMI may fail to identify up to half of individuals with excess percent fat mass [38]. Our results should stress how important it is to create preventive public policy, focusing on behavioral modifications irrespective of BMI, to react on the association of NWO with metabolic imbalances at this early age. Such implementations could limit further complications that may arise as these individuals get older.

## Supporting Information

S1 Dataset Raw data. (XLSX)

## References

[1] Anderssen SA, Cooper AR, Riddoch C, Sardinha LB, Harro M, Brage S, et al Low cardiorespiratory fitness is a strong predictor for clustering of cardiovascular disease risk factors in children independent of country, age and sex. Eur J Cardiovasc Prev Rehabil. 2007;14(4):526–31. 10.1097/HJR.0b013e328011efc1 . 17667643

[2] Berenson GS, Srinivasan SR, Bao W, Newman WP 3rd, Tracy RE, Wattigney WA. Association between multiple cardiovascular risk factors and atherosclerosis in children and young adults. The Bogalusa Heart Study. N Engl J Med. 1998;338(23):1650–6. 10.1056/NEJM199806043382302 . 9614255

[3] Olafsdottir E, Aspelund T, Torfadottir JE, Steingrimsdottir L, Sigurdsson G, Thorsson B, et al [Early life residency associated with the risk of developing type 2 diabetes—the population-based Reykjavik study]. Laeknabladid. 2012;98(12):639–44. . 23232723 10.17992/lbl.2012.12.466

[4] Strom M, Halldorsson TI, Mortensen EL, Torp-Pedersen C, Olsen SF. Fish, n-3 fatty acids, and cardiovascular diseases in women of reproductive age: a prospective study in a large national cohort. Hypertension. 2012;59(1):36–43. 10.1161/HYPERTENSIONAHA.111.179382 . 22146511

[5] Magnussen CG, Smith KJ, Juonala M. When to prevent cardiovascular disease? As early as possible: lessons from prospective cohorts beginning in childhood. Curr Opin Cardiol. 2013;28(5):561–8. 10.1097/HCO.0b013e32836428f4 . 23928921

[6] Barker DJ. The developmental origins of adult disease. J Am Coll Nutr. 2004;23(6 Suppl):588S–95S. . 15640511 10.1080/07315724.2004.10719428

[7] Morrison JA, Glueck CJ, Woo JG, Wang P. Risk factors for cardiovascular disease and type 2 diabetes retained from childhood to adulthood predict adult outcomes: the Princeton LRC Follow-up Study. Int J Pediatr Endocrinol. 2012;2012(1):6 10.1186/1687-9856-2012-6 22507454 PMC3466140

[8] Zhu S, Wang Z, Shen W, Heymsfield SB, Heshka S. Percentage body fat ranges associated with metabolic syndrome risk: results based on the third National Health and Nutrition Examination Survey (1988–1994). Am J Clin Nutr. 2003;78(2):228–35. . 12885702 10.1093/ajcn/78.2.228

[9] Marchesini G, Bugianesi E, Forlani G, Cerrelli F, Lenzi M, Manini R, et al Nonalcoholic fatty liver, steatohepatitis, and the metabolic syndrome. Hepatology. 2003;37(4):917–23. 10.1053/jhep.2003.50161 . 12668987

[10] Kaur J. A Comprehensive Review on Metabolic Syndrome. Cardiol Res Pract. 2014;2014:943162 10.1155/2014/943162 . 24711954 PMC3966331

[11] De Lorenzo A, Martinoli R, Vaia F, Di Renzo L. Normal weight obese (NWO) women: an evaluation of a candidate new syndrome. Nutr Metab Cardiovasc Dis. 2006;16(8):513–23. 10.1016/j.numecd.2005.10.010 . 17126766

[12] Madeira FB, Silva AA, Veloso HF, Goldani MZ, Kac G, Cardoso VC, et al Normal weight obesity is associated with metabolic syndrome and insulin resistance in young adults from a middle-income country. PLoS One. 2013;8(3):e60673 Epub 2013/04/05. 10.1371/journal.pone.0060673 ; PubMed Central PMCID: PMCPmc3610876. 23556000 PMC3610876

[13] Romero-Corral A, Somers VK, Sierra-Johnson J, Korenfeld Y, Boarin S, Korinek J, et al Normal weight obesity: a risk factor for cardiometabolic dysregulation and cardiovascular mortality. Eur Heart J. 2010;31(6):737–46. Epub 2009/11/26. 10.1093/eurheartj/ehp487 ; PubMed Central PMCID: PMCPmc2838679. 19933515 PMC2838679

[14] Addo OY, Himes JH. Are field measures of adiposity sufficient to establish fatness-related linkages with metabolic outcomes in adolescents? Eur J Clin Nutr. 2014 10.1038/ejcn.2014.14 . 24569541

[15] Cerhan JR, Moore SC, Jacobs EJ, Kitahara CM, Rosenberg PS, Adami HO, et al A pooled analysis of waist circumference and mortality in 650,000 adults. Mayo Clin Proc. 2014;89(3):335–45. 10.1016/j.mayocp.2013.11.011 . 24582192 PMC4104704

[16] Olafsdottir AS, Thorsdottir I, Gunnarsdottir I, Thorgeirsdottir H, Steingrimsdottir L. Comparison of women's diet assessed by FFQs and 24-hour recalls with and without underreporters: Associations with biomarkers. Ann Nutr Metab. 2006;50(5):450–60. 10.1159/000094781. WOS:000240937800008. 16877864

[17] Arngrimsson SA, Petitt DS, Borrani F, Skinner KA, Cureton KJ. Hyperthermia and maximal oxygen uptake in men and women. Eur J Appl Physiol. 2004;92(4–5):524–32. Epub 2004/05/20. 10.1007/s00421-004-1053-1 . 15150660

[18] Hinriksdottir G, Tryggvadottir A, Olafsdottir AS, Arngrimsson SA. Fatness but Not Fitness Relative to the Fat-Free Mass Is Related to C-Reactive Protein in 18 Year-Old Adolescents. PLoS One. 2015;10(6):e0130597 Epub 2015/06/16. 10.1371/journal.pone.0130597 26075745 PMC4468067

[19] Borg GA. Psychophysical bases of perceived exertion. Med Sci Sports Exerc. 1982;14(5):377–81. Epub 1982/01/01. . 7154893

[20] Puavilai W, Laorugpongse D, Deerochanawong C, Muthapongthavorn N, Srilert P. The accuracy in using modified Friedewald equation to calculate LDL from non-fast triglyceride: a pilot study. J Med Assoc Thai. 2009;92(2):182–7. . 19253792

[21] Friedrich N, Thuesen B, Jorgensen T, Juul A, Spielhagen C, Wallaschofksi H, et al The association between IGF-I and insulin resistance: a general population study in Danish adults. Diabetes Care. 2012;35(4):768–73. 10.2337/dc11-1833 22374641 PMC3308317

[22] Lohman TG, Houtkooper LB, Going SB. Body fat measurements goes hi-tech: Not all are created equal. ACSM‘s Health Fitness J 1997;(1):30–5.

[23] Alberti KG, Eckel RH, Grundy SM, Zimmet PZ, Cleeman JI, Donato KA, et al Harmonizing the metabolic syndrome: a joint interim statement of the International Diabetes Federation Task Force on Epidemiology and Prevention; National Heart, Lung, and Blood Institute; American Heart Association; World Heart Federation; International Atherosclerosis Society; and International Association for the Study of Obesity. Circulation. 2009;120(16):1640–5. 10.1161/CIRCULATIONAHA.109.192644 . 19805654

[24] Marques-Vidal P, Pecoud A, Hayoz D, Paccaud F, Mooser V, Waeber G, et al Normal weight obesity: relationship with lipids, glycaemic status, liver enzymes and inflammation. Nutr Metab Cardiovasc Dis. 2010;20(9):669–75. Epub 2009/09/15. 10.1016/j.numecd.2009.06.001 . 19748248

[25] Oliveros E, Somers VK, Sochor O, Goel K, Lopez-Jimenez F. The concept of normal weight obesity. Prog Cardiovasc Dis. 2014;56(4):426–33. Epub 2014/01/21. 10.1016/j.pcad.2013.10.003 . 24438734

[26] Marques-Vidal P, Chiolero A, Paccaud F. Large differences in the prevalence of normal weight obesity using various cut-offs for excess body fat. E Spen Eur E J Clin Nutr Metab. 2008;3(4):e159–e62. 10.1016/j.eclnm.2008.05.003

[27] Conus F, Rabasa-Lhoret R, Peronnet F. Characteristics of metabolically obese normal-weight (MONW) subjects. Appl Physiol Nutr Metab. 2007;32(1):4–12. Epub 2007/03/03. 10.1139/h07-926 . 17332780

[28] Evans EM, Rowe DA, Racette SB, Ross KM, McAuley E. Is the current BMI obesity classification appropriate for black and white postmenopausal women? Int J Obes (Lond). 2006;30(5):837–43. Epub 2006/01/19. 10.1038/sj.ijo.0803208 . 16418761

[29] Pineau JC, Frey A. Comparison of skinfold thickness models with dexa: impact of visceral adipose tissue. J Sports Med Phys Fitness. 2015 Epub 2015/02/14. . 25678207

[30] Shea JL, King MT, Yi Y, Gulliver W, Sun G. Body fat percentage is associated with cardiometabolic dysregulation in BMI-defined normal weight subjects. Nutr Metab Cardiovasc Dis. 2012;22(9):741–7. Epub 2011/01/11. 10.1016/j.numecd.2010.11.009 . 21215604

[31] Mannisto S, Harald K, Kontto J, Lahti-Koski M, Kaartinen NE, Saarni SE, et al Dietary and lifestyle characteristics associated with normal-weight obesity: the National FINRISK 2007 Study. Br J Nutr. 2014;111(5):887–94. Epub 2013/11/16. 10.1017/s0007114513002742 . 24229475

[32] Conus F, Allison DB, Rabasa-Lhoret R, St-Onge M, St-Pierre DH, Tremblay-Lebeau A, et al Metabolic and behavioral characteristics of metabolically obese but normal-weight women. J Clin Endocrinol Metab. 2004;89(10):5013–20. Epub 2004/10/09. 10.1210/jc.2004-0265 . 15472199

[33] Choi J, Se-Young O, Lee D, Tak S, Hong M, Park SM, et al Characteristics of diet patterns in metabolically obese, normal weight adults (Korean National Health and Nutrition Examination Survey III, 2005). Nutr Metab Cardiovasc Dis. 2012;22(7):567–74. Epub 2010/12/28. 10.1016/j.numecd.2010.09.001 . 21186103

[34] Stallmann-Jorgensen IS, Gutin B, Hatfield-Laube JL, Humphries MC, Johnson MH, Barbeau P. General and visceral adiposity in black and white adolescents and their relation with reported physical activity and diet. Int J Obes (Lond). 2007;31(4):622–9. Epub 2007/03/27. 10.1038/sj.ijo.0803587 . 17384663

[35] Hill JO, Wyatt HR, Peters JC. Energy balance and obesity. Circulation. 2012;126(1):126–32. Epub 2012/07/04. 10.1161/circulationaha.111.087213 22753534 PMC3401553

[36] Miech RA, Kumanyika SK, Stettler N, Link BG, Phelan JC, Chang VW. Trends in the association of poverty with overweight among US adolescents, 1971–2004. JAMA. 2006;295(20):2385–93. 10.1001/jama.295.20.2385 . 16720824

[37] Deshmukh-Taskar PR, Nicklas TA, O'Neil CE, Keast DR, Radcliffe JD, Cho S. The relationship of breakfast skipping and type of breakfast consumption with nutrient intake and weight status in children and adolescents: the National Health and Nutrition Examination Survey 1999–2006. J Am Diet Assoc. 2010;110(6):869–78. 10.1016/j.jada.2010.03.023 . 20497776

[38] Pedersen SD, Astrup AV, Skovgaard IM. Reduction of misclassification rates of obesity by body mass index using dual-energy X-ray absorptiometry scans to improve subsequent prediction of per cent fat mass in a Caucasian population. Clin Obes. 2011;1(2–3):69–76. Epub 2011/04/01. 10.1111/j.1758-8111.2011.00016.x . 25585571

[39] Moy FM, Loh DA. Cardiometabolic risks profile of normal weight obese and multi-ethnic women in a developing country. Maturitas. 2015;81(3):389–93. Epub 2015/05/20. 10.1016/j.maturitas.2015.04.011 . 25987469

[40] Nelson MC, Story M, Larson NI, Neumark-Sztainer D, Lytle LA. Emerging adulthood and college-aged youth: an overlooked age for weight-related behavior change. Obesity (Silver Spring). 2008;16(10):2205–11. Epub 2008/08/23. 10.1038/oby.2008.365 . 18719665

[41] Alberga AS, Sigal RJ, Goldfield G, Prud'homme D, Kenny GP. Overweight and obese teenagers: why is adolescence a critical period? Pediatr Obes. 2012;7(4):261–73. Epub 2012/03/31. 10.1111/j.2047-6310.2011.00046.x . 22461384

[42] Jean N, Somers VK, Sochor O, Medina-Inojosa J, Llano EM, Lopez-Jimenez F. Normal-weight obesity: implications for cardiovascular health. Curr Atheroscler Rep. 2014;16(12):464 Epub 2014/10/25. 10.1007/s11883-014-0464-7 . 25342492

